# The Clinical Frailty Scale and incidence of adverse outcomes in older patients with hip fractures in Qatar

**DOI:** 10.3389/fmed.2025.1643181

**Published:** 2025-07-29

**Authors:** Shirmila Syamala, Francisco José Tarazona-Santabalbina, Jorge Luis Passarelli, Brijesh Sathian, Navas Nadukkandiyil, Hanadi Al Hamad

**Affiliations:** ^1^Department of Geriatrics and Long-Term Care, Hamad Medical Corporation, Doha, Qatar; ^2^Geriatric Medicine Department, Hospital Universitario de la Ribera, Alzira, Spain; ^3^Medical School, Universitat Catòlica de València Sant Vicent Màrtir, Valencia, Spain

**Keywords:** frailty, Clinical Frailty Scale, delirium, mortality, Qatar, Middle East, hip fracture, length of stay

## Abstract

**Background:**

Studies conducted on Western populations have shown that the Clinical Frailty Scale (CFS) is a major predictor of adverse outcomes in older patients with hip fractures; however, there are no data on Middle Eastern populations, who may be culturally and ethnically different. We examined the association between the preoperative Clinical Frailty Scale and multiple adverse outcomes in a cohort of patients with hip fractures (aged 60–96 years) in Qatar.

**Methods:**

This prospective, single-center observational cohort study included 155 patients aged ≥ 60 years with hip fractures from Qatar. These patients underwent a Clinical Frailty Scale assessment at baseline and were followed to evaluate four outcomes of interest: incident delirium, postoperative complications, all-cause mortality within a year, and increased length of stay (LoS) (LoS ≥ 14 days).

**Results:**

A total of 155 patients with hip fractures (average age 74.6 years, 46.5% women) were included in the study. At baseline, 72.2% had a Clinical Frailty Scale score of <5, 12.3% had a score of 5, and 15.5% had a score > 5. Higher baseline scores on the Clinical Frailty Scale were strongly and positively associated with delirium, postoperative complications, and all-cause mortality, but there was no association with length of hospital stay. Compared to the patients with Clinical Frailty Scale scores < 5, those with scores > 5 had significantly higher multivariable risk ratios (RR) (with 95% confidence interval [CI]) for various outcomes. Specifically, the RR for delirium was 7.76 (3.17–18.97), for postoperative complications, it was 3.59 (1.20–10.77), for all-cause mortality, it was 6.39 (1.45–28.20), and for length of stay ≥14 days, it was 1.43 (0.75–2.73).

**Conclusion:**

The Clinical Frailty Scale was positively associated with delirium, postoperative complications, and all-cause mortality but not with length of hospital stay in patients with hip fractures from Qatar.

## Introduction

Frailty is a well-recognized syndrome characterized by a decline in physiological and functional reserves in older adults, causing increased vulnerability to adverse health outcomes, even with minor stressors (1). As the global population is aging and increasingly requires complex medical and surgical care, the importance of screening for and addressing frailty in medical and surgical patients assumes great significance.

Frailty assessment is recommended in several pre-operative risk assessment guidelines (2), and various tools for the assessment of frailty have been utilized and show a strong correlation with outcomes (3). The Clinical Frailty Scale (CFS), a nine-point global assessment tool for frailty based on clinical assessments, was developed from extensive Canadian studies on frailty (4). The CFS is an accurate, reliable, and feasible instrument for preoperative frailty assessment (3). It can be completed in an average of 45 s after a preoperative clinical assessment and is logistically easy to perform (5). However, despite the availability of effective and feasible frailty assessment tools, their incorporation in the preoperative evaluation of older patients remains suboptimal (6, 7).

Hip fracture is an increasing public health challenge. According to the International Osteoporosis Foundation, there were 1.6 million patients with hip fractures globally in 2000, and this number is projected to increase up to 6.3 million by 2050 (8, 9). The incidence rate of osteoporotic hip fractures in Qatar has been reported as 141.7 per 100,000 for the population aged 50 years and older (10). Preoperative frailty has been associated with poor outcomes after hip fracture (11, 12). A higher Clinical Frailty Scale score has been found to be associated with adverse outcomes such as delirium, postoperative complications, mortality, and increased length of hospital stay in surgical patients (3, 13, 14). However, the majority of these studies were conducted on Western populations. Despite an estimated pooled frailty prevalence of 35% among older adults in the Middle East (15), no studies have examined the association between Clinical Frailty Scale scores and hip fracture outcomes in this region. In this context, we examined the association between the Clinical Frailty Scale and several adverse outcomes, including incident delirium during hospitalization, postoperative complications, all-cause mortality, and increased length of hospital stay, in a prospective sample of older adult patients with hip fractures from the largest healthcare facility in Qatar.

## Methods

This study was designed as a prospective, single-center observational cohort study. Consecutive inpatients aged ≥60 years who were residents of Qatar and admitted with neck of femur fractures between 2022 and 2024 at Hamad Medical Corporation—the largest acute tertiary care academic public sector hospital in Qatar—were included. Patients who refused to consent, were temporary visitors, had fractures resulting from high-impact trauma (e.g., traffic accidents), suffered from periprosthetic fractures, or were terminally ill were excluded. Informed consent was obtained from all patients (or their legally authorized representatives), and ethical approval for the study was obtained from the Hamad Medical Corporation Institutional Review Board (Reference number IRGC 05-JI-18-297; approved June 22, 2020).

Eligible patients were screened for inclusion in the study when they were admitted to the emergency department. Informed written consent was obtained to assess and follow up on patient outcomes, including electronic healthcare record reviews. Data collectors, including the study research assistant, pathway coordinator, and physical therapists, received training prior to the start of the study. Of the 252 patients with hip fractures screened during the study period, 42 were non-residents of Qatar on temporary visits, 45 refused to give consent, 2 had terminal illnesses, and 8 had high-impact trauma or periprosthetic fractures. A total of 155 patients with hip fractures who provided informed consent were included in the current study.

### Measures

All patients were assessed using the Clinical Frailty Scale (CFS), which is scored from 1 to 9, with 1 indicating ‘very fit’ and 9 indicating ‘terminally ill’ (4). Information was also collected on demographic variables, comorbidities, medications, length of stay (LoS), postoperative complications, and mortality up to 1 year. All patients underwent a comprehensive geriatric assessment conducted by the orthogeriatric team. Demographic and clinical variables, including diagnoses of diabetes, chronic kidney disease, and polypharmacy, were collected from hospital records.

To account for pre-existing diseases and medical conditions present at the time of admission, the Charlson Comorbidity Index was calculated. Patients were classified based on the scores calculated using the weighted categories of the CCI. Myocardial infarction, peripheral vascular disease, congestive heart failure, cerebrovascular disease, dementia, chronic obstructive pulmonary disease, peptic ulcer disease, mild liver disease, and uncomplicated diabetes mellitus were each assigned a score of one. Diabetes mellitus with end-organ damage, severe chronic kidney disease (on dialysis, status post-kidney transplant), solid tumors without metastasis, leukemia, and lymphoma were each assigned a score of two points. Moderate and severe liver diseases were assigned a score of three. Solid tumors with metastasis and AIDS were each assigned a score of six (16). Polypharmacy was defined as the use of five or more concurrent systemic medications (17).

Delirium was defined as an acute disturbance in attention and cognition that cannot be better explained by a pre-existing neurocognitive disorder, according to the DSM-V criteria. Screening for delirium was performed by the study team using the validated 4 AT score (18) and confirmed clinically by the team geriatrician. Our outcome measure was the new onset of delirium during hospitalization among study participants who were delirium-free at baseline.

The research team monitored the patients during their inpatient stay for postoperative complications. Post-operative complications included chest infection, surgical site infection, deep vein thrombosis and/or pulmonary embolism, bleeding, renal insufficiency, and pressure ulcers. Electronic medical records and death records were reviewed to identify mortality data for up to 1 year. An increased length of stay (LoS) was defined as a hospital stay of ≥14 days.

### Statistical analysis

We reported continuous data as means and standard deviations (SDs) and categorical data as counts with relative frequencies. A chi-squared test was performed to compare categorical variables between the groups. We categorized the CFS scores at baseline into three groups based on clinical relevance and available sample size: <5 (no frailty or vulnerable), 5 (mild frailty), and >5 (moderate, severe, or very severe frailty) (4). We also examined the Clinical Frailty Scale as a continuous variable. We calculated risk ratios (RRs) and 95% confidence intervals (CIs) for the association between Clinical Frailty Scale categories and our four outcomes of interest (incident delirium, in-hospital postoperative complications, all-cause mortality, and length of hospital stay ≥14 days), employing log-binomial regression models (19, 20) due to the short and relatively uniform duration of follow-up. We used an age-and sex-adjusted model and a multivariable-adjusted regression model, adjusting for age (years), sex (male, female), diabetes mellitus (yes, no), chronic kidney disease (yes, no), serum hemoglobin levels (mg/dL), polypharmacy (yes, no), and the Charlson Comorbidity Index (score). All analyses were conducted using R version 4.0.2.

## Results

Table 1 shows the baseline characteristics of the elderly study population. A total of 155 patients with hip fractures were included in our study, with an average age of 74.6 years (ranging between 60 and 96 years). Of these, 28.4% were aged 60–69 years, 44.5% were 70–79 years, and 27.1% were 80 years or older; 53.5% of the participants were men. At baseline, 69.7% had a clinical diagnosis of diabetes mellitus, 27.1% had chronic kidney disease, and 77.4% were on polypharmacy. The mean serum hemoglobin level was 11.8 mg/dL, and the mean Charlson Comorbidity Index score was 4.6. Regarding the Clinical Frailty Scale score distribution at baseline, 72.2% had a score of <5, 12.3% had a score of 5, and 15.5% had a score of >5.

**Table 1:** Characteristics of the study population.

Baseline characteristics	Number	Mean (SD) or %
Age, years	155	74.6 (7.6)
Age groups, %		
60–69 years	44	28.4%
70–79 years	69	44.5%
≥80 years	42	27.1%
Sex, %		
Male	83	53.5%
Female	72	46.5%
Diabetes mellitus, %		
Yes	108	69.7%
No	47	30.3%
Chronic kidney disease, %		
Yes	42	27.1%
No	113	72.9%
Serum hemoglobin, mg/dL	155	11.8 (1.9)
Polypharmacy, %		
Yes	120	77.4%
No	35	22.6%
Charlson Comorbidity Index, score	155	4.6 (1.6)
Clinical frailty scale categories, %		
<5	112	72.2%
5	19	12.3%
>5	24	15.5%

Incidence of adverse outcomes	Number	%
Delirium, %		
Yes	26	16.8%
No	129	83.2%
In-hospital postoperative complications, %^*^		
Yes	17	11.3%
No	133	88.7%
All-cause deaths, %		
Yes	9	5.8%
No	146	94.2%
Length of stay ≥14 days, %		
Yes	46	30%
No	109	70%

^*^Analysis confined to *n* = 150 out of the 155 patients who underwent hip fracture surgery.

Table 1 also shows the four outcomes of interest: incident delirium during hospital stay occurred in 16.8% of the patients; in-hospital postoperative complications occurred in 11.3%; all-cause mortality occurred in 5.8% during the follow-up period of up to 1 year; and the length of hospital stay was ≥14 days in 30% of patients. The length of hospital stay in this study ranged from 3 to 163 days, with a median value of 9 days. The cutoff of ≥14 days to define a prolonged hospital stay was determined by dividing the length of stay variable into tertiles and selecting the highest third as the outcome.

**Figure 1:**
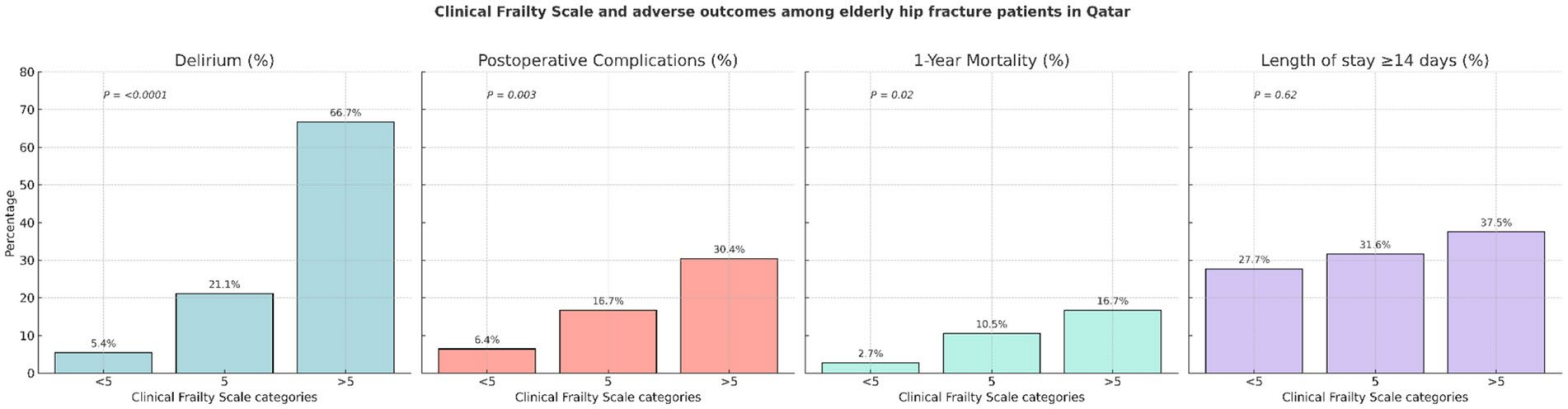
Clinical Frailty Scale and adverse outcomes among the older patients with hip fractures in Qatar.

Figure 1 shows the incidence of outcomes of interest by categories of the Clinical Frailty Scale at baseline divided into three groups: <5, 5, and >5. In separate analysis for each outcome, there was a clear pattern of statistically significantly higher incidence of delirium (*p <* 0.0001), in-hospital postoperative complications (*p* = 0.003), and all-cause mortality (*p* = 0.02) with increasing CFS groups. For example, compared to the participants with a Clinical Frailty Scale score < 5 (incidence = 5.4%), the incidence of delirium was almost 4 times higher in those with a Clinical Frailty Scale score of 5 (incidence = 21.1%) and more than 12 times higher in those with a Clinical Frailty Scale score of >5 (incidence = 66.7%). A similar pattern was observed in the analyses of postoperative complications (e.g., incidence of 6.3% vs. 29.2% comparing Clinical Frailty Scale categories <5 vs. >5) and all-cause mortality (incidence of 2.7% vs. 16.7% comparing Clinical Frailty Scale categories <5 vs. >5) as outcomes (see Figure 1).

However, as shown in Figure 1, there was no significant association between increasing Clinical Frailty Scale categories and longer hospital stay, which was defined as length of hospital stay ≥14 days (*p* = 0.62). In a supplementary analysis (data not presented in tables), first, we examined the mean length of stay by the Clinical Frailty Scale categories (<5, 5, and >5); the mean length of stay for these respective categories was 12.1 days, 19.7 days, and 17.9 days, and there was no statistically significant difference (*p* = 0.1367). Second, when we examined both length of stay (dependent variable) and the Clinical Frailty Scale score (independent variable) as continuous variables in a multivariable linear regression model, the association remained statistically non-significant (beta coefficient for Clinical Frailty Scale = 0.009; *p* = 0.7).

Tables 2–5 show the association between baseline Clinical Frailty Scale categories and incident delirium (Table 2), in-hospital postoperative complications (Table 3), all-cause mortality (Table 4), and length of stay ≥14 days (Table 5). For all outcomes, we examined the Clinical Frailty Scale at baseline as a 3-level variable (<5, 5, and >5) and a binary variable defined as the presence of clinical frailty at baseline (<5, ≥5). In the age-and sex-adjusted models and the multivariable-adjusted model, we found that higher baseline Clinical Frailty Scale categories and the binary clinical frailty variable were strongly and positively associated with delirium (Table 2), postoperative complications (Table 3), and all-cause mortality (Table 4). The overall pattern and direction of association for these outcomes were consistent and statistically significant. In contrast, there was no significant association between the baseline Clinical Frailty Scale categories or the binary clinical frailty variable and prolonged hospital stay, defined as length of stay ≥14 days (Table 5).

**Table 2:** Clinical Frailty Scale categories and incidence of delirium in the older patients with hip fractures.

Clinical Frailty Scale (CFS)	No. of participants (no. of delirium cases)	Percentage (%)	Age-and sex-adjusted risk ratio (95% confidence interval) of incident delirium	Multivariable-adjusted risk ratio (95% confidence interval) of incident delirium†
CFS categories^*^				
<5	112 (6)	5.4%	1 (Referent)	1 (Referent)
5	19 (4)	21.1%	3.53 (1.17–10.67)	3.45 (1.25–9.58)
>5	24 (16)	66.7%	10.15 (4.22–24.40)	7.76 (3.17–18.97)
*P*-trend			<0.0001	<0.0001
CFS continuous variable^*^	155 (26)	16.8%	2.19 (1.66–2.88)	1.98 (1.48–2.64)

^*^Clinical Frailty Scale based on Rockwood et al. (4). ^†^Multivariable log-binomial regression model adjusted for age (years), sex (male, female), diabetes mellitus (yes, no), chronic kidney disease (yes, no), serum hemoglobin levels (mg/dL), polypharmacy (yes, no), and the Charlson Comorbidity Index (score).

**Table 3:** Clinical Frailty Scale categories and incidence of postoperative complications in older patients with hip fractures.

Clinical Frailty Scale (CFS)	No. of participants (no of postoperative complication cases)	Percentage (%)	Age-and sex-adjusted risk ratio (95% confidence interval) of in-hospital postoperative complications	Multivariable-adjusted risk ratio (95% confidence interval) of in-hospital postoperative complications†
CFS categories^*^				
<5	109 (7)	6.4%	1 (Referent)	1 (Referent)
5	18 (3)	16.7%	2.25 (0.69–7.34)	2.34 (0.69–7.90)
>5	23 (7)	30.4%	3.40 (1.21–9.60)	3.59 (1.20–10.77)
*P*-trend			0.02	0.02
CFS continuous variable^*^	150 (17)	11.3%	1.40 (1.02–1.94)	1.42 (1.01–2.00)

^*^Clinical Frailty Scale based on Rockwood et al. (4). ^†^Multivariable log-binomial regression model adjusted for age (years), sex (male, female), diabetes mellitus (yes, no), chronic kidney disease (yes, no), serum hemoglobin levels (mg/dL), polypharmacy (yes, no), and the Charlson Comorbidity Index (score).

**Table 4:** Clinical Frailty Scale categories and all-cause mortality up to 1-year follow-up in older patients with hip fractures.

Clinical Frailty Scale	No. of participants (no. of deaths)	Percentage (%)	Age-and sex-adjusted risk ratio (95% confidence interval) of all-cause mortality	Multivariable-adjusted risk ratio (95% confidence interval) of all-cause mortality†
CFS categories^*^				
<5	112 (3)	2.7%	1 (Referent)	1 (Referent)
5	19 (2)	10.5%	2.64 (0.45–15.43)	3.06 (0.54–17.51)
>5	24 (4)	16.7%	6.01 (1.44–25.03)	6.39 (1.45–28.20)
*P*-trend			0.01	0.01
CFS continuous variable^*^	155 (9)	5.8%	1.77 (1.03–3.03)	1.83 (1.07–3.13)

^*^Clinical Frailty Scale based on Rockwood et al. (4). ^†^Multivariable log-binomial regression model adjusted for age (years), sex (male, female), diabetes mellitus (yes, no), chronic kidney disease (yes, no), serum hemoglobin levels (mg/dL), polypharmacy (yes, no), and the Charlson Comorbidity Index (score).

**Table 5:** Clinical Frailty Scale categories and length of stay ≥ 14 days in the older patients with hip fractures.

Clinical Frailty Scale (CFS)	No. of participants (length of stay ≥ 14 days)	Percentage (%)	Age-and sex-adjusted risk ratio (95% confidence interval) of length of stay ≥ 14 days	Multivariable-adjusted risk ratio (95% confidence interval) of length of stay ≥ 14 days†
CFS categories^*^				
<5	112 (31)	27.7%	1 (Referent)	1 (Referent)
5	19 (6)	31.6%	1.02 (0.49–2.12)	1.13 (0.51–2.50)
>5	24 (9)	37.5%	1.28 (0.69–2.37)	1.43 (0.75–2.73)
*P*-trend			0.47	0.32
CFS continuous variable^*^	155 (46)	29.7%	1.02 (0.83–1.24)	1.04 (0.84–1.29)

^*^Clinical Frailty Scale based on Rockwood et al. (4). ^†^Multivariable log-binomial regression model adjusted for age (years), sex (male, female), diabetes mellitus (yes, no), chronic kidney disease (yes, no), serum hemoglobin levels (mg/dL), polypharmacy (yes, no), and the Charlson Comorbidity Index (score).

In a second supplementary analysis (data not presented in tables) to study the relation between Clinical Frailty Scale categories and age, we performed a cross-tabulation of age categories (<80 years, ≥80 years) by Clinical Frailty Scale groups (<5, 5, >5). There was a statistically significant association between increasing Clinical Frailty Scale categories and older age. The percentage of participants aged ≥80 years was 20.5% among those with a score of <5, 42.1% among those with a score of 5, and 45.8% among those with a score of >5 (*p* = 0.01).

## Discussion

In this prospective cohort study of patients aged 60 years and older with hip fractures from Qatar, the Clinical Frailty Scale score at admission was positively associated with incident delirium, postoperative complications, and all-cause mortality, but it did not show an association with length of stay. Our results contribute to the existing literature on frailty and adverse outcomes by providing one of the few data sets on this topic from the Middle East. Our findings are largely consistent with reports from across the world (3, 11, 12, 21–25).

Frailty has been consistently associated with delirium in post-surgical and hip fracture patients in several studies (3, 5). In a systematic review of eight studies (n = 5,541, mean age 77.8), a 2.2-fold increased risk of delirium in persons with frailty was noted (24). Our results showed a similar association between higher Clinical Frailty Scale scores and delirium in patients with hip fractures. Frailty has been shown to be a state of low-grade inflammation, and the concept of “inflammaging” has been proposed (26), with inflammatory biomarkers such as IL-10, soluble TNF-*α* receptors, and ICAM-1 suggested as frailty markers (27). Neuroinflammation and cerebral metabolic insufficiency are the likely mechanisms underlying the pathophysiology of delirium in the context of frailty (23). Fracture and surgery can trigger an increase in systemic inflammatory mediators (28, 29), which are transported to the brain across the blood–brain barrier and through the transporters in the afferent nerves of the vagus nerve. Increased central inflammatory mediators can cause cerebral dysfunction through the suppression of hippocampal plasticity and neurogenesis, neurotoxicity, and neuronal apoptosis, all of which are implicated in the development of delirium (30–32). In addition, metabolic abnormalities such as alterations in the levels of glycolysis products, low serum lipid metabolic phosphatidylinositol, and increased serum neuropeptide galanin levels may predispose individuals with frailty to delirium, and the latter could be a potential biomarker for predicting postoperative delirium (23).

An association between frailty and mortality after surgical procedures has been well established (3, 13, 21, 33). Furthermore, studies have shown an increased risk of both short-term and long-term mortality in older patients with pre-existing frailty presenting with hip fractures (11, 22). Forssten et al. (11), in a Swedish nationwide retrospective study, reported a 4 times higher risk of mortality in patients with frail hip fractures. Narula et al. (25) reported on the predictive value of the Clinical Frailty Scale for mortality outcomes following proximal femur fractures. Alterations in innate and adaptive immunity with increased susceptibility to infections, impaired nutritional status, a heightened inflammatory response, and prolonged sympathetic activation with stressors such as fracture and surgery could be the possible mechanisms leading to multiple organ dysfunction and mortality in frail older adults (34–37).

A wealth of evidence suggests an association between frailty and adverse postoperative complications (3, 5, 25). In a study from the US, Kistler et al. (12) reported higher rates of postoperative complications and prolonged length of stay for patients with hip fractures using a modified frailty index. Frailty is hypothesized to be due to dysregulated stress response systems, including immune, endocrine, and energy response systems (38). Traditionally, preoperative risk assessments focused mainly on cardiovascular, anesthesia, and surgical risks; current guidelines recommend including frailty assessment for older individuals (2, 39). While the opportunity exists for prehabilitation to address frailty in elective surgeries, in geriatric patients with hip fractures, the healthcare team should focus on immediate perioperative risk intervention strategies based on orthogeriatric comprehensive care (23) and multi-component care bundles (40) to prevent postoperative delirium and other adverse outcomes.

Studies have shown both positive (13, 41) and negative (42) associations between frailty and length of stay. Vainqueur et al. (42) reported that in a retrospective cohort of 158 patients, frailty did not show a significant association with length of stay. Similarly, frailty did not show a significant association with length of stay in our group of Middle Eastern patients either, possibly because of early discharge to rehabilitation or other local cultural or sociodemographic factors affecting length of stay.

Our study is a confirmatory study in a new setting (i.e., Middle Eastern population) examining the association between frailty and adverse outcomes in patients with hip fractures. In this group of patients aged 60 years and above presenting with hip fractures, 27.9% were assessed to have frailty at admission—12.3% were assessed to be living with mild frailty (CFS of 5) and 15.6% with moderate to severe frailty (CFS 6 and above), based on the Clinical Frailty Scale. This is slightly lower than the prevalence of frailty reported in other studies in patients with hip fractures, which ranged between 41 and 53% (11, 33, 43). This could be attributed to the different frailty assessment tools used and the relatively younger age of our study population, as our cutoff age was 60 years and above. Even so, our data further validate and extend the evidence supporting the importance of frailty screening using the Clinical Frailty Scale in this vulnerable population from the Middle Eastern region.

This study has some limitations. First, we would like to acknowledge that our findings may be prone to a certain degree of selection bias due to the exclusion of certain individuals—specifically, 21.7% of eligible individuals who refused consent—which may have potentially resulted in an overestimation of the findings. Second, the sample size, while reasonable, was underpowered for some outcomes (e.g., only nine deaths), leading to wide confidence intervals, limiting the robustness of the findings—particularly for the mortality analysis (44). However, the overall pattern and positive direction of the association remained consistent even after adjusting for confounders. Furthermore, a similar strong association between frailty and delirium has been reported in other studies (45, 46). Similarly, the lack of a significant association for length of stay—despite a clinically meaningful trend in average length of stay by the Clinical Frailty Scale, could be due to limited statistical power. Therefore, our findings of no associations for length of stay need to be confirmed in larger, well-powered studies from the Middle East. Third, although we adjusted for potential associated factors in the multivariable analysis, the effects of unmeasured confounding factors, such as nutritional status, caregiver support, socioeconomic status, dementia, the American Society of Anesthesiologists (ASA) classification score, time to surgery, and fracture pattern, may have influenced the results. Finally, we did not perform direct comparisons between the Clinical Frailty Scale and other frailty assessment tools, such as the FRAIL scale or frailty phenotype; however, comparing multiple frailty scales was not the primary objective of the current study. An advantage of this study is that our sample is representative of all patients with hip fractures in Qatar, as Hamad Medical Corporation is the major government-funded tertiary care hospital managing orthopedic emergencies nationwide.

In conclusion, frailty as measured by the Clinical Frailty Scale showed a strong positive association with adverse outcomes, including incident delirium during hospitalization, in-hospital postoperative complications, and all-cause mortality, but not with length of hospital stay in a cohort of older patients with hip fractures from Qatar. The Clinical Frailty Scale is a simple and feasible tool that should be incorporated into the preoperative assessment of older adults. Studies with larger sample sizes from the Middle East are needed to confirm and also expand on our findings.

## Data Availability

The datasets presented in this article are not readily available because of patient confidentiality. Further inquiries can be directed to the corresponding authors. Requests to access the datasets should be directed to SS, ssyamala@hamad.qa.
